# The Relationship between Osteoinduction and Vascularization: Comparing the Ectopic Bone Formation of Five Different Calcium Phosphate Biomaterials

**DOI:** 10.3390/ma15103440

**Published:** 2022-05-10

**Authors:** Yun He, Yu Peng, Lishuang Liu, Sha Hou, Junyu Mu, Liang Lan, Lijia Cheng, Zheng Shi

**Affiliations:** College of Basic Medicine, Clinical Medical College, Affiliated Hospital of Chengdu University, Chengdu 610106, China; heyun@cdu.edu.cn (Y.H.); pengyu1216@163.com (Y.P.); cheery.lau@hotmail.com (L.L.); mujunyu@stu.cdu.edu.cn (S.H.); mjy2373541378@163.com (J.M.); lan2103934375@163.com (L.L.)

**Keywords:** hydroxyapatite, tricalcium phosphate, osteoinduction, vascularization, nanoscale

## Abstract

**Objective**: The objective of this study is to compare the bone induction of five kinds of calcium phosphate (Ca-P) biomaterials implanted in mice and explore the vascularization and particle-size-related osteoinductive mechanism. **Methods:** The following five kinds of Ca-P biomaterials including hydroxyapatite (HA) and/or tricalcium phosphate (TCP) were implanted in the muscle of 30 BALB/c mice (*n* = 6): 20 nm HA (20HA), 60 nm HA (60HA), 12 µm HA (12HA), 100 nm TCP (100TCP) and 12 µm HA + 100 nm TCP (HATCP). Then, all animals were put on a treadmill to run 30 min at a 6 m/h speed each day. Five and ten weeks later, three mice of each group were killed, and the samples were harvested to assess the osteoinductive effects by hematoxylin eosin (HE), Masson’s trichrome and safranine–fast green stainings, and the immunohistochemistry of the angiogenesis and osteogenesis markers CD31 and type I collagen (ColI). **Results:** The numbers of blood vessels were 139 ± 29, 118 ± 25, 78 ± 15, 65 ± 14 in groups HATCP, 100TCP, 60HA and 20HA, respectively, which were significantly higher than that of group 12HA (12 ± 5) in week 5 (*p* < 0.05). The area percentages of new bone tissue were (7.33 ± 1.26)% and (8.49 ± 1.38)% in groups 100TCP and HATCP, respectively, which were significantly higher than those in groups 20HA (3.27 ± 0.38)% and 60HA (3.43 ± 0.27)% (*p* < 0.05); however, no bone tissue was found in group 12HA 10 weeks after transplantation. The expression of CD31 was positive in new blood vessels, and the expression of ColI was positive in new bone tissue. **Conclusions:** Nanoscale Ca-P biomaterials could induce osteogenesis in mice muscle, and the osteoinductive effects of TCP were about 124% higher than those of 20HA and 114% higher than those of 60HA. The particle size of the biomaterials affected angiogenesis and osteogenesis. There was a positive correlation between the number of blood vessels and the area percentage of new bone tissue; therefore, osteoinduction is closely related to vascularization. Our results provide an experimental basis for the synthesis of calcium–phosphorus matrix composites and for further exploration of the osteoinductive mechanism.

## 1. Introduction

In addition to autologous and allogeneic bone grafting, artificial bone grafting has become a promising method for filling bone defects caused by fracture, congenital osteogenesis imperfecta, bone neoplasms and trauma that greatly improves the quality of life of orthopedic patients. The osteoinductive phenomenon of calcium phosphate (Ca-P) biomaterials at non-bone sites of animals has been known for more than 30 years [1,2], and Ca-P biomaterials have been widely used in the field of artificial bone grafting. At present, the osteoinductive mechanism of Ca-P biomaterials is not well understood. In terms of cell regulation, Ca-P biomaterials could mediate bone integration by stimulating osteoclasts. The appropriate Ca/P ratio in materials could effectively promote RANKL–RANK binding and activate more NF-κB signal transduction, leading to osteoclast differentiation. Ca-P biomaterials also increase the gene expression of several growth factors in macrophages, including insulin-like growth factor (IGF), epidermal growth factor (EGF), transforming growth factor β-1 (TGFβ-1) and vascular endothelial growth factor (VEGF), which might influence the behavior of bone marrow mesenchymal stem cells (BMSCs), in addition to the currently accepted research theory of protein adsorption and ion concentration [3,4,5]. Relatively speaking, the chemical composition, mechanical strength, pore structure and morphology of macroscopic materials, as well as animal species and humoral factor of transplanted animals, are the best controlled factors affecting osteogenesis [6]. Of all the factors that affect osteogenesis, the elements, morphology and particle size of the material itself are very important factors. Ca-P materials themselves are not bone-induced; the bone-induced properties of Ca-P materials can be introduced in two ways: (1) design and appropriate geometry combined with appropriate porosity; (2) Ca-P binds to growth factors or bioactive proteins, but it is still affected by the pores, composition and particle size of Ca-P biomaterials [7]. The nature of bone induction requires Ca-P to have specific pore and particle sizes. The most commonly used Ca-P biomaterials mainly include hydroxyapatite (HA), beta-tricalcium phosphate (β-TCP) and biphasic calcium phosphate (BCP), composed of different ratios of HA and β-TCP. The crystallinity, porosity, nanostructure, particle size, percentage of HA and β-TCP of Ca-P biomaterials are various factors affecting osteogenesis. In order to achieve excellent osteogenic effects, these materials are usually synthesized through sintering at over 1000 °C [8,9,10,11]. In fact, powdery materials without sintering and pore structure could also induce bone neoformation [12,13]. Therefore, we focus on the particle size of powdery HA or TCP to explore the effect of particle size on vascularization and osteogenesis.

With the development of nanotechnology, the biomaterials being fabricated are smaller and smaller, and nanoscale HA is being applied. Nanoscale HA was added to polymer materials to make an ε-poly-L-lysine (EPL)-coated nanoscale polycaprolactone/hydroxyapatite (EPL/PCL/HA) composite that showed enhanced bone repair capacity [14]; a highly interweaved thiolated hyaluronic acid-nano-hydroxyapatite (HA-SS-nHAp)/collagen hybrid fibering hydrogel significantly promoted osteoinductivity and mineralization compared with composite combined micro-hydroxyapatite [15]; another study showed that nano-HA exhibited a significant amount of bone regeneration, including lots of woven and lamellar bone, compared with that of forstrite scaffolds in dog premolar defects [16]. On the contrary, nano-TCP has barely been reported. In our study, we compare the osteogenic abilities of micro-HA and nano-HA, and those of nano-HA and nano-TCP; we also explore the relative mechanism of osteoinduction triggered by Ca-P biomaterials.

## 2. Materials and Methods

### 2.1. X-ray Diffraction (XRD)

X-ray diffraction (XRD) measurements were performed using an X-ray diffractometer (Bruker D8 ADVANC; Germany) operating at 40 kV and 40 mA. Diffractograms were measured over the range of 2-Theta (θ) from 10° to 80° with an angular step interval of 0.020°. The standard cards (No. 97-009-9358 and No. 97-005-6310) had the highest matching degree with HA and TCP after phase analysis using Jade9.0 software (Santa Clara, CA, USA).

### 2.2. Scanning Electron Microscopy (SEM)

A scanning electron microscopy (SEM) analysis of the samples was performed using a Hitachi S4800 field-emission instrument (Tokyo, Japan). The SEM images were measured in backscattered electron mode (BSE) at ambient vacuum. The sample was sprayed with vacuum gold for 10 min. The accelerating voltage used was 5 KV. Image acquisition under vacuum conditions and magnification used were from 1 K to 100 K.

### 2.3. Animal Surgery

Thirty 8-week-old male BALB/c mice were obtained from Dossy Biological Technology Company (Chengdu, China). All animals were maintained in a temperature- and light-controlled environment ventilated with filtered air. All animals were randomly divided into five groups (*n* = 6). During surgery, mice were anaesthetized with an intraperitoneal injection of 3% pentobarbital sodium (1 mg/100 g body weight). Hair on the thigh was removed using an electric shaver, and the skin underneath was disinfected with ethanol; then, an approximately 10 mm longitudinal skin incision was made parallel to the femur. After that, an approximately 8 mm longitudinal muscle pouch was prepared immediately along the skin incision. Next, 10 μg of powder material was weighed and wetted with sterile PBS before implantation; then, the same 10 μg of wetted 20 nm HA (20HA), 60 nm HA (60HA), 12 µm HA (12HA) and 100 nm TCP (100TCP), and a mixture of 5 μg of 12 µm HA and 5 μg of 100 nm TCP (HATCP) biomaterial were implanted into the muscle pouches of the left and right legs, respectively, which were far away from the femur. At last, the incised muscle and skin were closed with nylon sutures, and 40 mg penicillin was injected intramuscularly to prevent infection. Then, all animals were put on a treadmill to run 30 min at a 6 m/h speed each day. The mice were killed with the cervical dislocation method, and the samples were harvested 5 w and 10 w after implantation. The study was approved by the Animal Care and Use Committee of Chengdu University. The operative procedures and animal care were performed in compliance with the NIH guidelines on the care and use of laboratory animals, under the supervision of a licensed veterinarian.

### 2.4. Histological Staining

Briefly, the harvested samples were cleaned with PBS three times, then fixed in 2.5% glutaraldehyde for approximately 24 h at room temperature. Subsequently, the samples were decalcified in 10% ethylenediaminetetraacetic acid (EDTA), pH 7.0, for about 20 days at room temperature; they were washed with diethyl pyrocarbonate (DEPC), then dehydrated using 50%, 70%, 85%, 95% and 100% ethanol solutions for 2 h each. Next, they were embedded in paraffin (melting point, 56–58 °C) and sliced into 5 µm thick histological sections using a microtome. Finally, hematoxylin eosin (HE), Masson’s trichrome and safranine–fast green (S&G) stainings were performed on the samples according to the manufacturer’s instructions. Images were then scanned and analyzed with a NanoZoomer Digital Pathology scanner (NDP; Hamamatsu, Japan).

### 2.5. Immunohistochemistry

The expression of CD31 and type I collagen (ColI) was analyzed by immunostaining. To block endogenous peroxidase, the sections were treated with 3% H_2_O_2_ for 15 min in the dark, rinsed three times with double-distilled water, then retrieved with Tris–EDTA buffer (pH 9.0) in a 95 °C water bath for 45 min. Next, the specimens were incubated with a mouse monoclonal antibody against CD31 (1:1000; Abcam, Boston, MA, USA), ColI (1:1000; Abcam), and VEGF (1:1000, Abcam), and a horseradish peroxidase (HRP)-labeled secondary antibody (1:1000; Abcam). Finally, they were developed with 3,3-diaminobenzidine (DAB) and counterstained with hematoxylin.

### 2.6. Morphological Analysis

Blood vessels and bone tissue were identified by two pathologists. The new blood vessels were counted and comparatively analyzed among the five groups using image-pro plus (IPP) software in week 5; meanwhile, the area of new bone and total tissue were encircled and automatic measured, and the area percentage of new bone was calculated as new bone area/total tissue area using the NanoZoomer Digital Pathology scanner’s built-in software in week 10. For the morphological analysis, three scanned HE slices were analyzed separately, and the number of the new vessels and the area percentage of new bone were the average of the three sections.

### 2.7. Statistical Analysis

Data are expressed as means ± standard deviation ($\bar{X}$ ± s); they were analyzed by the Student’s *t*-test and Pearson correlation analysis (SPSS 22.0; Chicago, IL, USA). A *p* < 0.05 was considered statistically significant.

## 3. Results

### 3.1. The XRD Spectra of Materials

**Figure 1:**
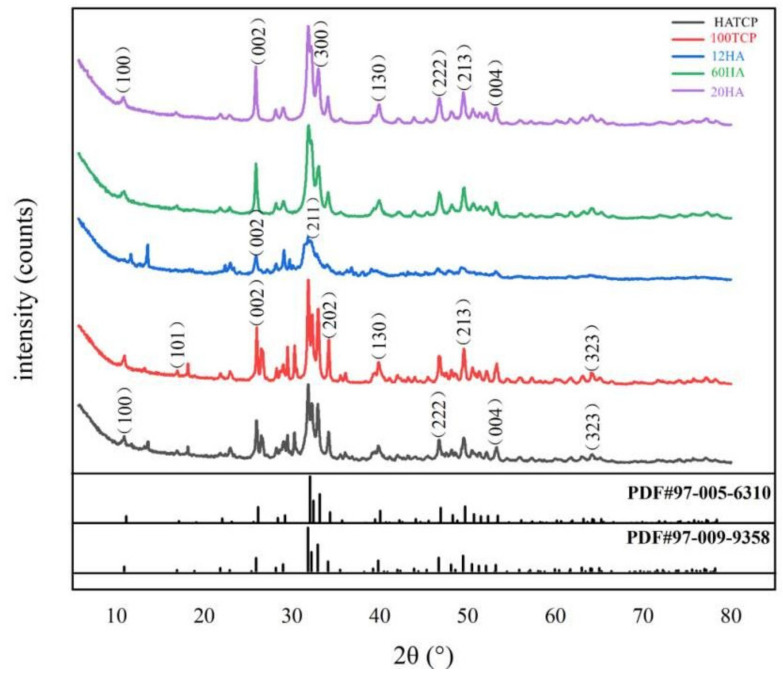
XRD patterns of 20HA, 60HA, 12HA, 100TCP and HATCP biomaterials, compared with the standard HA (No.97-009-9358) and TCP (No.97-005-6310).

The five kinds of Ca-P powder biomaterials were passed through a 320-mesh sieve with an aperture of 47 µm, and the chemical components were detected by XRD and analyzed by Jade 9.0 software. Figure 1 shows the XRD pattern of these materials; the peaks observed in the figure can be assigned to (100), (101), (002), (211), (300), (202), (130), (222), (213), (004) and (323) reflections of HA and TCP crystals. The diffraction patterns of 20HA, 60HA and 12HA were consistent with standard HA (No.97-009-9358) and the diffraction pattern of 100TCP was consistent with standard TCP (No.97-005-6310), whose peaks were different. As the particle size of HA increases, its peak decreases correspondingly. We can also see that HATCP has characteristic peaks of both HA and TCP. For example, in the 20°–40° section, we can see that HATCP has peaks similar to 100TCP and a characteristic peak of 12HA. The particle size of 20 nm HA and 60 nm HA matched the actual size calculated by the Debye–Scherrer formula.

### 3.2. The Micromorphology of Materials

**Figure 2:**
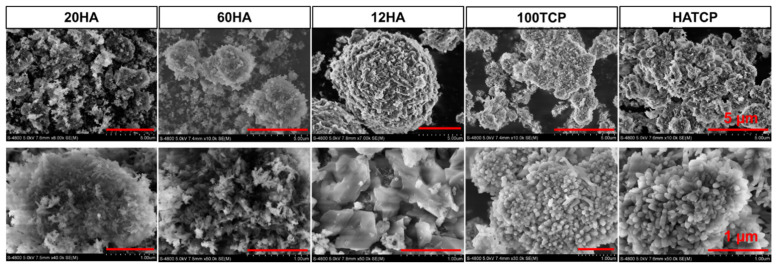
The SEM photos show the micromorphology of 20HA, 60HA, 12HA, 100TCP and HATCP biomaterials at two different magnifications. Bar above: 5 µm; bar below: 1 µm.

The five kinds of Ca-P powder biomaterials were passed through a 320-mesh sieve and scanned by SEM after gold spraying. Then, the morphological characteristics of the materials were analyzed according to the micro photos. The particle sizes and morphologies of the five groups of materials were completely different; the particles presented tip shapes in groups 20HA and 60HA, while the particles showed spheres in group 12HA; furthermore, group 100TCP showed thin cylinders, different from HA, and the material was a mixture of 12HA and 100TCP in group HATCP (Figure 2).

### 3.3. Angiogenesis Prior to Bone Formation

**Figure 3:**
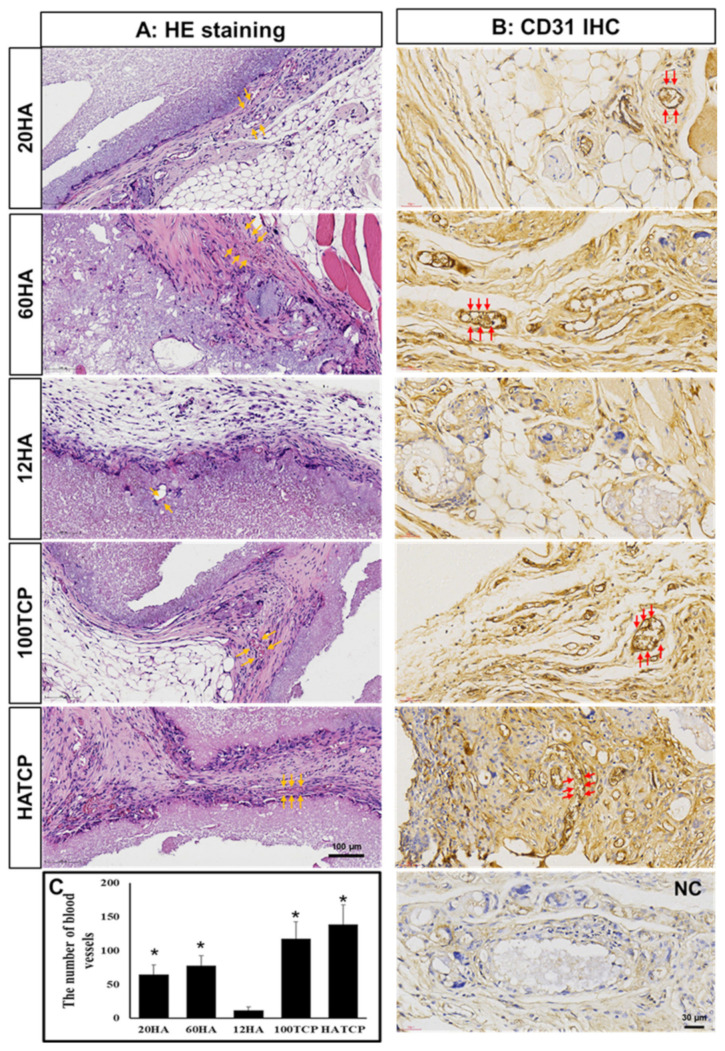
Angiogenesis observed following morphologic staining. (**A**) HE staining shows new-blood-vessel formation in groups 20HA, 60HA, 12HA, 100TCP and HATCP. Arrow: blood vessels; bar: 100 µm. (**B**) Immunohistochemical staining for CD31 shows that blood vessels were stained brown. NC: negative control; arrow: blood vessels; bar: 30 µm. (**C**) The number of blood vessels in the five groups was counted based on IPP software; * *p* < 0.05.

There was blood-vessel formation, but no bone formation, five weeks after transplantation. The number of blood vessels observed was different among the five groups. There were almost no new blood vessels in group 12HA due to mesenchymal tissue only being able to grow at the edge of the material, while lots of capillaries formed in the other four groups, because the mesenchymal tissue grew in the interior of the material (Figure 3A). Platelet endothelial cell adhesion molecule-1 (PECAM-1/CD31) was specifically expressed on vascular endothelial cells, and the immunohistochemical staining for CD31 (Figure 3B) and VEGF (Figure S1) was positive in the blood vessels. The number of blood vessels was the largest in group HATCP, and the number of blood vessels was significantly higher in the other four groups than that in group 12HA (*p* < 0.05 Figure 3C). Blood vessels can provide nutrients for new-bone formation, and the amount of blood-vessel formation is proportional to the amount of new-bone formation later observed.

### 3.4. Osteoinduction of Ca-P Biomaterials

**Figure 4:**
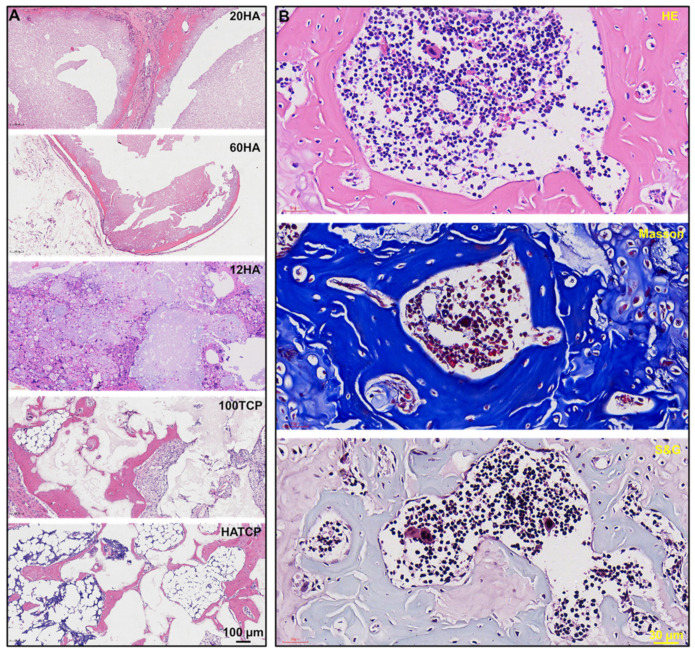
Osteoinduction of Ca-P biomaterials. (**A**) New-bone formation was observed in groups 20HA, 60HA, 100TCP and HATCP but not ingroup 12HA. Bar: 100 µm. (**B**) HE, Masson’s and safranine–fast green stainings of group HATCP showed that new bone tissues were pink, blue and light green. Bar: 30 µm.

**Figure 5:**
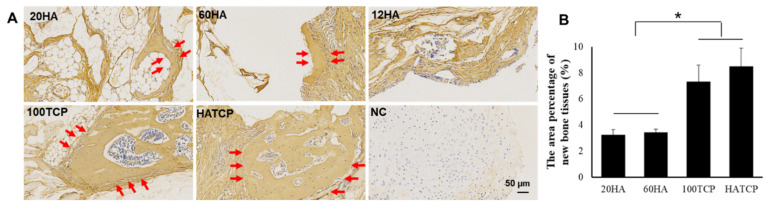
Immunohistochemistry of ColI and the quantitative analysis of new bone tissue. (**A**) Immunohistochemistry of ColI showed it was positively expressed in mineral bone. NC: negative control; arrow: bone tissue; bar: 50 µm. (**B**) The area percentage of new bone tissue in groups 20HA, 60HA, 100TCP and HATCP. * *p* < 0.05.

New-bone formation was observed in groups 20HA, 60HA, 100TCP and HATCP, but not ingroup 12HA, ten weeks after transplantation, and bone-marrow tissue was found in some samples of groups 100TCP and HATCP (Figure 4A). On a continuous section of an osteogenic specimen from group HATCP, HE, Masson’s and S&G stainings were performed; the results showed that mature lamellar bone and bone marrow tissue were detected, and the collagen fibers of bone tissue were stained pink, blue and light green following HE, Masson’s and S&G stainings, respectively (Figure 4B). The results of the immunohistochemical staining for ColI showed that ColI was identified in the newly formed bone tissue, while the un-degraded materials were nonspecifically stained (Figure 5A).

### 3.5. Quantitative Analysis of Bone Induction

**Figure 6:**
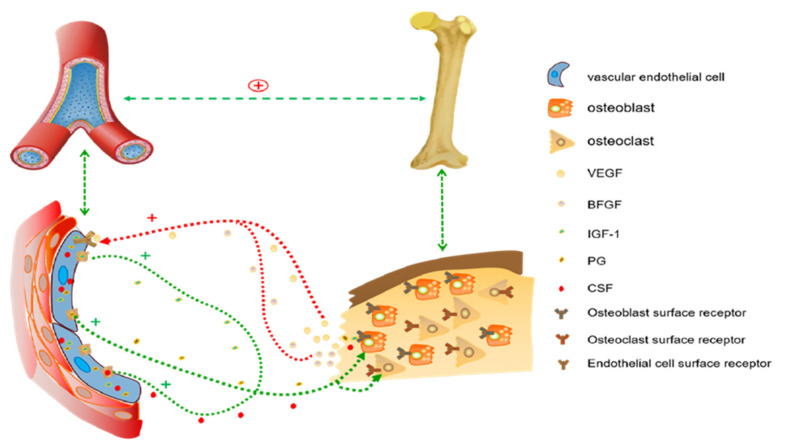
The schematic diagram shows the relationship between vascularization and osteogenesis.

The area percentage of new bone tissue was calculated as new bone area/total tissue area. The statistical results showed that the Ca-P biomaterials with TCP could induce more bone tissue, and that the area percentage of new bone tissue was significantly higher in groups 100TCP and HATCP than that in groups 20HA and 60HA (*p* < 0.05; Figure 5B). The osteoinductive effects of TCP were about 124% higher than those of 20HA and 114% higher than those of 60HA. Although 12 µm HA could not induce bone-tissue formation, it did stimulate TCP to form more bone tissue. The amount of new-bone formation was proportional to the number of blood vessels previously observed; the correlation between the number of blood vessels and the area percentage of new bone tissue is shown in Table 1; *p* < 0.05. There was a positive correlation between osteoinduction and vascularization. The mechanisms associated with angiogenesis and osteogenesis are shown in Figure 6.

## 4. Discussion

XRD and SEM were used to evaluate the crystallinity and structural properties of nano-sized Ca-P biomaterials. The material characteristics analyzed by SEM showed that the shapes of HA at the nanometer and micron scales were different, but the XRD confirmed that the two were consistent with the standard HA diffraction peak, which did not affect the crystallinity and properties of HA.

Biomaterial-induced bone formation is more likely to occur in large animals, such as dogs, goats, sheep and non-human primates. In the research design, we set up a bone-induction model for small animals based on the Ca-P biomaterial designed for traditional large animals, such as that by Li et al. for rabbit mandible repair [17,18]. The validation of our nanoaperture Ca-P biomaterial also further compensates for the difference in response of traditional bone induction materials in different individual populations and explores new ideas for the use of synthetic bone-graft substitutes for clinical research.

The bone conduction capacity of bone implants depends on complex interactions among material type, surface and microstructure. Ghayor et al. concluded that the microstructure of bone substitute materials had strong bone conduction based on aperture in the range 0.7~1.2 mm and bottleneck in the range 0.5~1.2 mm [19]. However, bone induction requires a much smaller aperture. In our study, the biomaterials were implanted into the muscle far away from the femur; although new-bone formation was caused by osteoinduction, bone conduction cannot be completely ruled out. Moreover, the good bone conductivity and bone inductivity of the material was finally reflected through the efficiency of new-bone formation and vascularization of the muscle pocket.

Calcium is a very important element during bone induction of biomaterials. We found that 20 nm HA, 60 nm HA and 100 nm TCP could induce new-bone formation in mice; however, calcium does not necessarily induce bone formation, since 12 µm HA did not induce any bone formation. In future studies, nanoscale HA can be used as bone grafting material. Usually, HA has higher stiffness and less degradability than TCP, in which Ca^2+^ can be replaced by a variety of metal ions through the ion-exchange reaction, forming corresponding metal-ion apatite; therefore, HA is commonly used as the surface coating of titanium alloys and other metals to prepare hard-tissue implant materials [20,21,22,23]. Although HA is osteoinducible, it has often been reported that it could not induce bone tissue formation on its own [24]. The reason has to do with the structure of the material and the animal species. Due to its material properties, HA has often been used in combination with other materials, osteogenic cells or osteogenic factors, such as collagen [25], polylactic acid (PLA) [26], sodium alginate (SA) [27], chitosan [28], graphene oxide [29], Zinc [30], BMSCs [31], bone morphogenetic protein-2 (BMP-2) [32], etc. Of course, HA has most commonly been used in conjunction with TCP, since TCP has better osteoinductive and degradation abilities [33,34]. In our study, TCP induced more bone formation than HA, and the combination of HA and TCP enhanced the osteogenesis of TCP. HA/TCP biomaterials are also called biphasic calcium phosphate (BCP) ceramics, which have proven efficacy in numerous clinical indications. Their specific physico-chemical properties, including the HA/TCP ratio, dual porosity and subsequent interconnected architecture, the progressive resorption and the bone substitution process, have been extensively reported [35]. HA/TCP has not become the gold standard for bone grafting, despite the efforts of scholars in studying the sintering temperature, porosity, composition ratio and other aspects. To achieve optimal bone repairing results, it is still necessary to combine Ca-P biomaterials with other osteogenic materials, cells and factors, i.e., relying on bone-tissue engineering.

Vascularization is the basis for new-bone formation by Ca-P biomaterials. Neovascularization can supply small-molecule nutrients to new bone tissue; therefore, new-blood-vessel formation is necessary during the process of ectopic bone formation [36]. Vascularization is an early event in bone induction, and angiogenesis can induce osteoclasts to enhance the absorption of old bone and promote the proliferation of osteoblasts to form new bone. The specific mechanism is that vascular endothelial cells can secrete IGF, prostaglandin and colony stimulating factor (CSF), which affect osteoblast differentiation. Osteoblasts can also promote VEGF, and basic fibroblast growth factor (BFGF) generate to regulate vascular endothelial cells, further promoting angiogenesis [37]. On the one hand, endothelial progenitor cells differentiate to form new blood vessels; on the other hand, existing blood vessels in the muscle of the mice form new capillaries by budding and fusing with their capillaries. In our study, numerous new blood vessels were observed five weeks after transplantation, and the number of blood vessels was reduced by week 10 based on HE staining, since significant new-bone formation happened after the absorption of nutrients provided by blood vessels. The number of new blood vessels was positively correlated with the area percentage of new bone tissue. The number of blood vessels in the HA/TCP mixed group was higher than that in the nanoscale-HA and -TCP groups. The combined effect of HA and TCP could promote the bone formation of TCP, and the formation of blood vessels provided nutrition for new-bone formation. Vascularization is an important mechanism of bone induction. Angiogenesis-related factors, such as VEGF, BFGF, can also be added to bone grafting materials to promote osteogenesis in bone-tissue engineering.

## 5. Conclusions

We compared the angiogenesis and osteogenesis of five kinds of Ca-P biomaterials, 20 nm HA, 60 nm HA, 12 µm HA, 100 nm TCP and 12 µm HA/100 nm TCP, and found that TCP or HATCP could promote more blood-vessel formation than HA by week 5; moreover, we also observed that nanoscale HA and TCP, but not micron-sized HA, could induce bone tissue formation; however, micron-sized HA could help TCP induce more bone tissue by week 10. Therefore, the particle size of biomaterials affected angiogenesis and osteogenesis. There was a positive correlation between the number of blood vessels and the area percentage of new bone tissue; therefore, osteoinduction is closely related to vascularization. In conclusion, bone induction is inseparable from vascularization, and blood vessels can transport nutrients for new-bone formation. Our results provide ideas for scientists to synthesize new materials and further explore osteoinductive mechanisms, and for clinicians to develop new treatments for orthopedic patients.

## Figures and Tables

**Table 1:** The correlation between the number of blood vessels and the area percentage of new bone tissue.

Groups	Number of Blood Vessels	Area Percentage of New Bone Tissue (%)	F	*p*
20HA	65	3.27	13.9327	0.0202
60HA	78	3.43		
12HA	12	0		
100TCP	118	7.33		
HATCP	139	8.49		

*p* < 0.05; Pearson correlation analysis of the five groups.

## Data Availability

Data in compliance with “MDPI Research Data Policies” at https://www.mdpi.com/ethics.

## Supplementary Materials

The following supporting information can be downloaded at: https://www.mdpi.com/article/10.3390/ma15103440/s1, Figure S1: IHC of VEGF in the five groups.
