# Supplementary material for: The Relationship between Osteoinduction and Vascularization: Comparing the Ectopic Bone Formation of Five Different Calcium Phosphate Biomaterials
Source: Materials (Basel). 2022 May 10;15(10):3440. doi: 10.3390/ma15103440 (PMC9146137; doi:10.3390/ma15103440)
Supplement: Supplementary file 1 [file materials-15-03440-s001.zip › materials-1669450-supplementary.pdf]

Supplementary materials

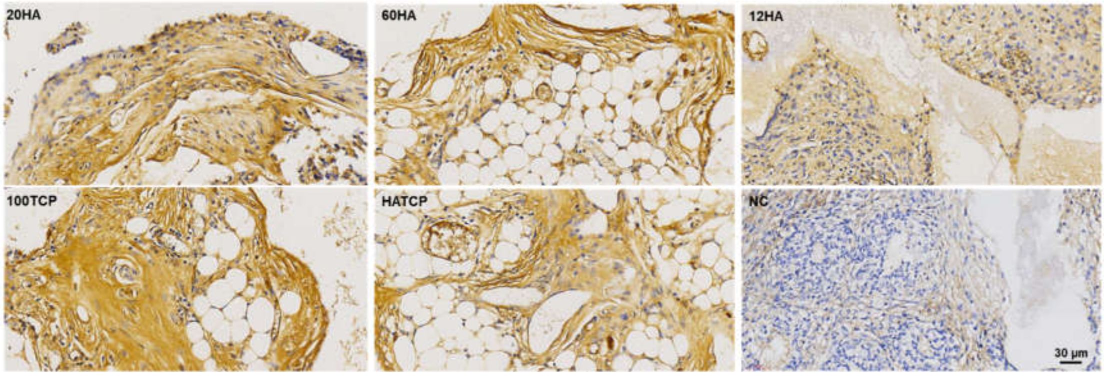

Supplementary Figure S1: IHC of VEGF in the five groups. The specimens of groups 20HA, 60HA, 12HA, 100TCP and HATCP were incubated with a mouse monoclonal antibody against vascular endothelial growth factor (VEGF, 1:1000, Abcam) and a horseradish peroxidase (HRP)-labeled secondary antibody (1:1000, Abcam). Finally, they were developed with 3,3-diaminobenzidine (DAB) and counterstained with hematoxylin. The results showed VEGF was positive expressed in new blood vessels. NC: negative control; bar: 30 μm.
